# Myeloid neoplasms with isolated isochromosome 17q demonstrate a high frequency of mutations in *SETBP1, SRSF2, ASXL1* and *NRAS*

**DOI:** 10.18632/oncotarget.7350

**Published:** 2016-02-12

**Authors:** Rashmi Kanagal-Shamanna, Rajyalakshmi Luthra, Cameron C. Yin, Keyur P. Patel, Koichi Takahashi, Xinyan Lu, John Lee, Chong Zhao, Francesco Stingo, Zhuang Zuo, Mark J. Routbort, Rajesh R. Singh, Patricia Fox, Farhad Ravandi, Guillermo Garcia-Manero, L. Jeffrey Medeiros, Carlos E. Bueso-Ramos

**Affiliations:** ^1^ Department of Hematopathology, The University of Texas MD Anderson Cancer Center, Houston, TX, USA; ^2^ Department of Leukemia, The University of Texas MD Anderson Cancer Center, Houston, TX, USA; ^3^ Department of Statistics, The University of Texas MD Anderson Cancer Center, Houston, TX, USA

**Keywords:** myeloid neoplasms, isochromosome 17q, SETBP1, SRSF2, ASXL1

## Abstract

Isolated isochromosome 17q, i(17q), accounts for less than 1% of myeloid neoplasms that are commonly classified as myelodysplastic/myeloproliferative neoplasms, acute myeloid leukemia (AML), myelodysplastic syndrome (MDS) or myeloproliferative neoplasms (MPN). We have shown previously that these cases have distinctive clinicopathologic features, a poor prognosis and absence of *TP53* mutations. However, their molecular mutation profile has not been studied. Here, we explored the mutation profile of 32 cases of myeloid neoplasm with isolated i(17q) that included AML, MDS/MPN, MDS and MPN. In addition to the common i(17q), these neoplasms had frequent mutations in *SRSF2* (55%), *SETBP1* (59%), *ASXL1* (55%), and *NRAS* (31%); *TET2* and *TP53* mutations were rare. Eight of 28 patients (29%) showed concurrent mutations in *ASXL1, SRSF2, SETBP1* and *RAS*. There was a significant association between mutations in *SETBP1* and RAS (*p* = 0.003). The mutation pattern was independent of the morphologic diagnosis. Sequential analysis of 5 cases showed evolution from a diploid karyotype to i(17q) and that *SRSF2* and *ASXL1* mutations precede the detection of i(17q) whereas *SETBP1* mutations are associated with i(17q).

## INTRODUCTION

Isochromosome 17q [i(17q)] is a non-random cytogenetic abnormality that involves deletion of the short “p” arm and duplication of the long “q” arm of chromosome 17. It is frequently observed in the setting of a complex karyotype in blast phase of chronic myelogenous leukemia (CML) and acute myeloid leukemia (AML). As an isolated cytogenetic abnormality, isochromosome (17q) in myeloid malignancies is rare with less than 150 reported cases. The presence of isolated i(17q) in myeloid neoplasms is associated with certain distinctive clinicopathologic findings as shown in several recent studies [1–3]. These neoplasms are often classified as myelodysplastic/myeloproliferative neoplasms (MDS/MPN) or high-grade myelodysplastic syndrome (MDS)/AML, and show characteristic morphologic findings, in particular pseudo Pelger-Huet neutrophils and small hypolobated megakaryocytes. Irrespective of the diagnosis or blast count at presentation, myeloid neoplasms with isolated i(17q) have an aggressive clinical course. Most patients undergo rapid progression to AML, often without acquisition of additional clonal cytogenetic abnormalities. Accordingly, i(17q) is classified as “intermediate” cytogenetic prognostic subgroup per the revised International Prognostic Scoring System (IPSS) for MDS [4, 5]; and as an “adverse” cytogenetic prognostic subgroup per revised Medical Research Council classification for AML [6].

Understanding the molecular alterations in myeloid neoplasms associated i(17q) will likely prove to be valuable in the development of targeted therapy for affected patients. The “low-copy repeats” rich breakpoint region, located at 17p11.2 is highly unstable and prone to be affected by large-scale genomic alterations including uniallelic loss of *TP53* [7, 8]. Until now, studies have shown that the presence of i(17q) abnormality is associated with wild-type *TP53* [1] and mutations in *SETBP1* and *SRSF2* [3, 9]. However, the molecular consequences of i(17q) are largely unknown. Further, it is unclear if the i(17q) abnormality precedes these gene mutations or represents a secondary event.

In this study, we performed a systematic molecular analysis of myeloid neoplasms with isolated i(17q) and discovered unique molecular alterations that provide insights into underlying pathogenesis and potential therapeutic targets. Using sequential mutation analysis in 5 cases that showed evolution of i(17q) abnormality from a diploid karyotype, we show that *SRSF2* and *ASXL1* mutations precede the detection of i(17q), whereas *SETBP1* mutations are associated with i(17q).

## RESULTS

We selected 32 cases of myeloid neoplasm with i(17q) as the primary abnormality that had sufficient DNA for molecular analysis. This group included 13 cases of MDS/MPN, 17 cases of AML (5 of which had a history of myeloid neoplasm), and 1 case each of MDS and MPN (clinical data shown in Table 1). Twenty-nine cases had i(17q) as a sole abnormality (2 acquired +13 subsequently during the course of the disease), 2 had 1 additional abnormality and 1 had multiple additional abnormalities. All cases were negative for BCR/ABL1 rearrangement.

**Table 1:** Summary of clinicopathologic and cytogenetic findings of 32 myeloid neoplasms with isolated i(17q) cytogenetic abnormality

Variable	MDS/MPN (*n* = 13, 41%)	AML (*n* = 17, 53%)	MDS (*n* = 1, 3%)	MPN (*n* = 1, 3%)
Median age (range)	64 years (51–83)	66 years (24–78)	55 years	60 years
Gender (female/male)	9/4	8/9	0/1	0/1
Hemoglobin* (g/dL)	9.4 (6.8–12.1)	10.1 (6.4–12.8)	8.6	12.9
MCV	88 (78–102)	88 (70–110)	91.5	104
Absolute neutrophil count*, × 10^9^/L	3.6 (0.5–122.2)	3.0 (0–24.2)	1.9	5.7
Platelet count*, × 10^9^/L	54 (10–143)	57 (15–271)	92	141
Peripheral blood blasts* (%)	1 (0–13)	26 (0–97)	0	1
Bone marrow blasts* (%)	8 (2–19)	37 (7–93)	12	0
Cytogenetic evolution, *n* (%)	30.8%	23.5%		
Without clonal evolution	9	13	0	1
With clonal evolution	4	4	1	0
Survival	12 died, 1 lost to follow-up	14 died, 1 alive, 2 lost to follow-up	Died	Died
2008 WHO sub-classification	CMML (*n* = 6) MDS/MPN-U (*n* = 5) aCML (*n* = 2)	*De novo*/AML MRC (*n* = 9); sAML: h/o myeloid neoplasm (*n* = 5) and therapy-related (*n* = 2); AML, relapsed post-allogeneic SCT, unclassifiable (*n* = 1)	RAEB-2	Post-ET MF

aCML, atypical chronic myeloid leukemia; AML, acute myeloid leukemia; AML MRC, AML with myelodysplasia-related changes; CMML, chronic myelomonocytic leukemia; MCV, mean corpuscular volume; MDS, myelodysplastic syndrome; MDS/MPN-U, myelodysplastic/myeloproliferative neoplasm unclassifiable; Post-Et MF, Post-essential thrombocythemia myelofibrosis; RAEB, refractory anemia with excess blasts; sAML, secondary AML; SCT, stem cell transplant.

**Figure 1:**
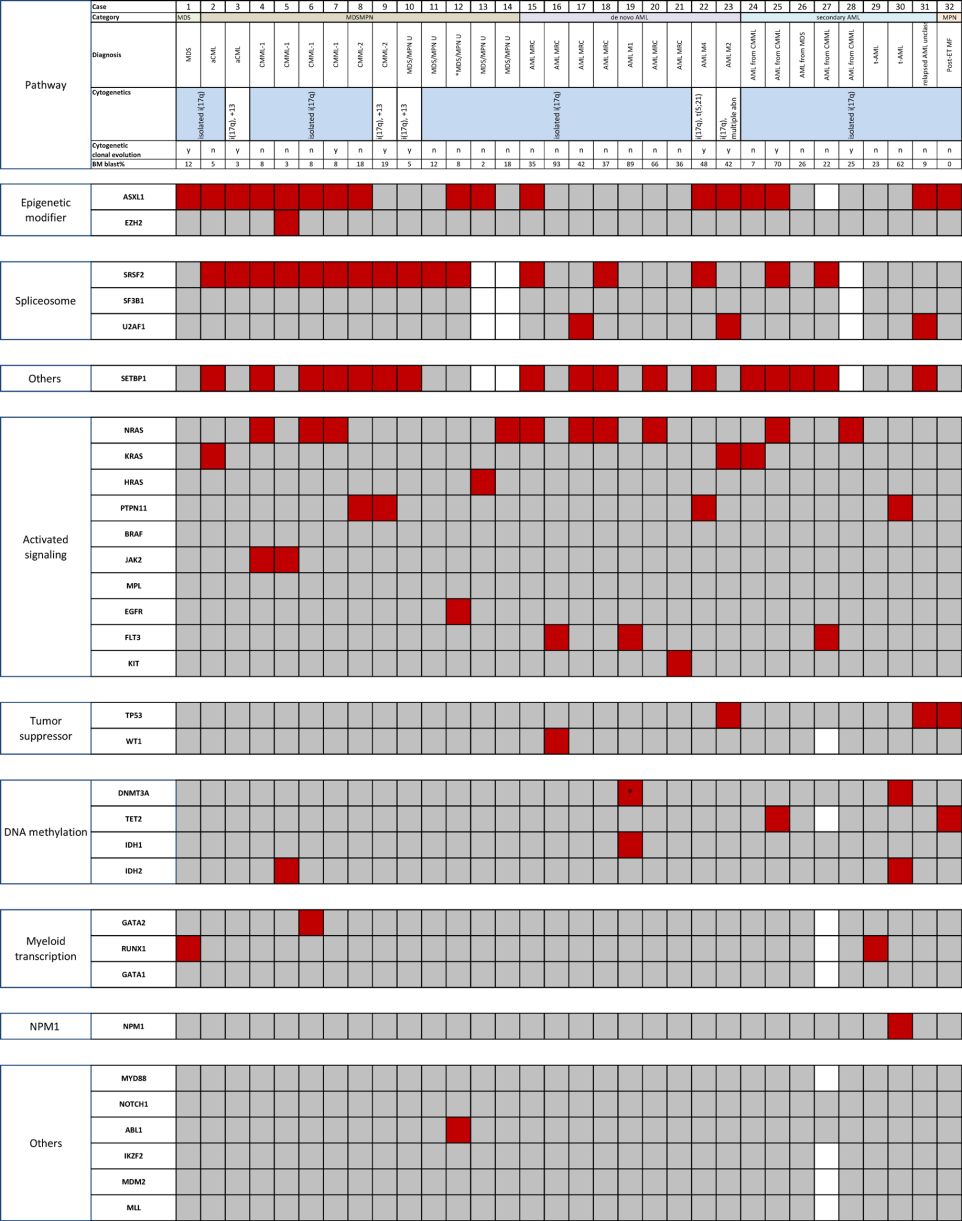
Mutational analysis of 32 cases of myeloid neoplasm with isolated i(17q) (red, mutation; gray, wild-type; white, not tested) Genes are segregated based on the biologic functional categories on the left. The upper panels denote the case number, diagnostic categories per 2008 WHO classification and clonal cytogenetic abnormalities. Case #31 could not be sub-classified. Isochromosome (17q) was detected on a post-allogeneic stem cell transplant sample that showed relapsed AML. The pre-transplant cytogenetic studies were diploid, and molecular studies were unavailable. The patient also had a history of breast carcinoma treated with chemotherapy. Cases 7, 9 and 31 (highlighted in lavender) also underwent whole-exome sequencing. *represents a non-R882 *DNMT3A* mutation. aCML, atypical chronic myeloid leukemia; AML, acute myeloid leukemia; AML MRC, AML with myelodysplasia-related changes; CMML, chronic myelomonocytic leukemia; ET, Essential thrombocythemia; MDS, myelodysplastic syndrome; MPN, myeloproliferative neoplasm; MDS/MPN, myelodysplastic/myeloproliferative neoplasm; MDS/MPN-U, myelodysplastic/myeloproliferative neoplasm unclassifiable; MF, myelofibrosis; RAEB, refractory anemia with excess blasts; sAML, secondary AML; SCT, stem cell transplant.

We analyzed three cases with isolated i(17q) using whole-exome sequencing and found mutations in genes that included *SETBP1*, *SRSF2*, and *ASXL1* (Supplementary Table 1). We performed mutation analysis of 33 genes using a combination of next-generation sequencing based analysis on all 32 cases targeting the coding regions of 28 genes; and Sanger sequencing for splicing factors, *CEBPA, MDM4* and *SETBP1*. The mutation results are shown in Figure 1. The details are provided in Supplementary Table 2. The median number of mutations per sample was 3. Genes with the highest frequency of mutations included: *SETBP1* (17/29; 59%), *ASXL1* (17/31; 55%), *SRSF2* (16/29; 55%), and *NRAS* (10/32; 31%). Mutations in genes that directly affect the *RAS* pathway was noted in 18 (56%) cases and included: *NRAS* (*n* = 10, 31%), *PTPN11* (*n* = 4; 13%), *KRAS* (*n* = 3; 9%), and *HRAS* (*n* = 1; 3%). These four mutations were mutually exclusive with each other, and in all but 1 case, they were exclusive with other genes affecting RAS/MAPK signaling pathway including *EGFR, FLT3, KIT*, and *BRAF*, but not *SETBP1*. There was a significant association between mutations in *SETBP1* and *RAS* (*p* = 0.003). There was a trend towards association between mutations in *SETBP1* and *SRSF2* (*p* = 0.07); and *SETBP1* and *ASXL1* (*p* = 0.07). Eight of 28 patients (29%) showed concurrent mutations in *ASXL1, SRSF2, SETBP1* and *RAS*. Ten patients (35%) showed concurrent mutations in *SRSF2*, *SETBP1* and *RAS*. Notably, mutations in *TET2*, *TP53* and *U2AF1* were rare. *SF3B1* mutations were absent. *SETBP1* and *TP53* mutations were mutually exclusive as reported by others [3, 9, 10] except in 1 case where *TP53* mutation was observed at 5% variant allelic frequency (VAF) in a post allogeneic stem cell transplant setting. In addition to the findings shown in Figure 1, none of the tested cases had mutations in *CEBPA* (*n* = 15) or *MDM4* (*n* = 12).

Segregation of genes based on the functional classification per Cancer Genome Atlas Research Network showed that mutations occurred in highest frequencies in genes involved in activating signaling pathways (mostly *RAS*), followed by splicing factor-encoding and epigenetic modifier genes (Supplementary Figure 1) [11]. Of the 4 most frequently mutated genes, VAF could be estimated for the genes of *RAS* pathway and *ASXL1* as these were analyzed by NGS. The median VAF of *NRAS* and *ASXL1* mutations in our cohort were 41.7 (range, 5–49.2) and 32.8 (range, 6–72) respectively. Eleven cases showed mutations in both *RAS* and *ASXL1*. In 8 of these cases, the VAF of *RAS* mutation was either similar to or higher than of *ASXL1*. The VAFs of *SETBP1* and *SRSF2* could only be assessed in 3 cases that underwent whole exome sequencing. The results showed that all mutated genes had a similar VAF in all cases.

The median overall survival (OS) from the onset of disease was 22.1 months, and median OS from the onset of i(17q) was 9.4 months (Kaplan-Meier curves shown in Supplementary Figure 3). There were no apparent differences in the mutation profile between the cases of AML and MDS/MPN, although the numbers are too small for significance.

**Figure 2:**
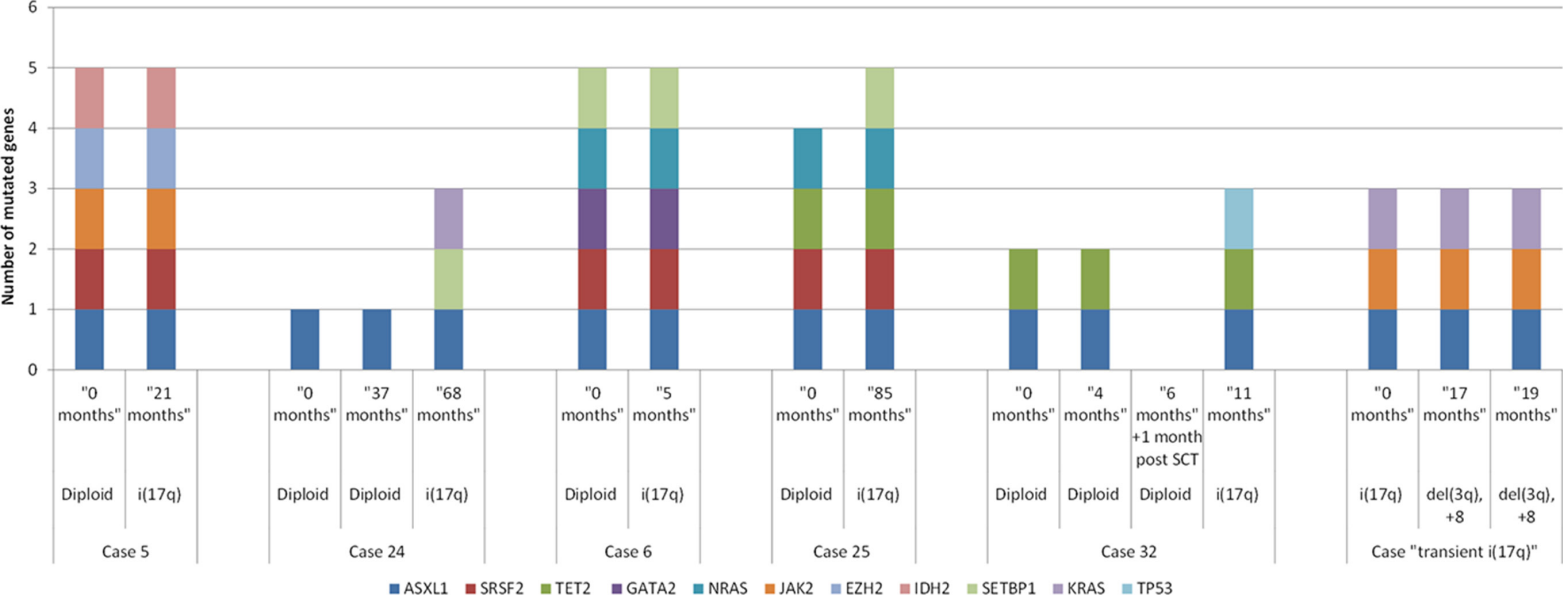
Sequential mutation analysis of myeloid neoplasms during the evolution from a diploid karyotype to the development of isolated i(17q) abnormality In case #5, i(17q) was observed after allogeneic stem cell transplant. In case #6, i(17q) abnormality was transient (noted for only a duration of 6 months).

Within this study cohort, 5 cases had an initial diploid karyotype and subsequently acquired i(17q). We performed mutation analysis on these patients at various time points during the evolution from diploid to karyotype showing i(17q) (as shown in Figure 2). Within the interval between the initial analysis to the development of i(17q) abnormality, all patients had undergone treatment with drugs that included decitabine, Ruxolitinib, hydroxyurea, dasatinib and lenalidomide. In one case, the i(17q) abnormality was acquired post-allogeneic stem cell transplantation. Mutations in *ASXL1, SRSF2* and *RAS* appear stable over time. *ASXL1* mutations were present in all 5 cases at both diploid and i(17q) stages of karyotypic evolution. *SRSF2* mutations were also present at both diploid and i(17q) stages in 3 of 5 patients. Mutations in *NRAS* (*n* = 2) and *PTPN11* (*n* = 1) at high allelic frequencies (> 35%) were also stable, being present at the diploid and i(17q) stages. In 3 patients, additional mutations were observed at the time of i(17q) abnormality. These mutations occurred in *SETBP1* (*n* = 2), *KRAS* (*n* = 1, subclonal, 7%) and *TP53* (*n* = 1, subclonal, 5%; post allogeneic stem cell transplant) genes. The only case that showed *SETBP1* mutation at the time of diploid karytoype developed i(17q) within 5 months.

## DISCUSSION

Myeloid neoplasms associated with isolated i(17q) show characteristic clinicopathologic features and aggressive behavior. In order to study molecular aberrations associated with the presence of i(17q), and to exclude the confounding effects of additional karyotypic aberrations, we included cases showing i(17q) as a sole karyotypic abnormality and performed a comprehensive mutational analysis. The genes with the highest mutation frequency included *ASXL1, SRSF2, SETBP1* and *RAS* signaling pathway genes, noted in more than 50% of the samples. In most of our cases, *RAS* mutations were detected at high allelic frequencies with a median of 42.1%. We performed pyrosequencing on majority of cases to confirm *RAS* mutations. These results have clinical implications because patients with myeloid neoplasms associated with i(17q) have a poor outcome and *RAS* and splicing factor mutations provide potential targets for treatment.

We also observed a high frequency of *ASXL1, SETBP1* and *SRSF2* mutations in myeloid neoplasms associated with i(17q). Similar observations were presented by Meggendorfer et al. at the 2015 ASH meeting (abstract# 1656; session: 636. Myelodysplastic Syndromes – Basic and Translational Studies: Poster I) [12]. However, the frequency of *RAS* mutations reported by the authors was much lower. This discordance may be related to the differences in the patient cohort, as their study included 63 patients, of which only 27 had isolated i(17q). The remainder of patients in their study had additional abnormalities including some patients with a complex karyotype.

Mutations in *ASXL1* (3/9, 33%), *SRSF2* (3/9, 33%), and *SETBP1* (5/9, 56%) were also observed in *de novo* AML cases (*n* = 9). Lindsley et al. have shown that mutations in *ASXL1* and splicing factors, as observed in our cohort, are highly specific for secondary AML [13]. Gene mutations in *NPM1, DNMT3A, CEBPA, IDH1* and *FLT3*, that are reportedly common in *de novo* AML, were infrequent. Thus, the presence of i(17q) may indicate an underlying MDS or MDS/MPN, at least in a subset of AML cases, even in *de novo* setting. The only case with an *NPM1* mutation was a patient with relapsed AML status post allogeneic stem cell transplant. The patient had a history of breast carcinoma treated with chemotherapy and AML with a diploid karyotype. Cytogenetic studies showed i(17q) in 7 of 20 metaphases. In the presence of *NPM1* mutation, i(17q) abnormality may have been transiently acquired due to the damaging effects of chemotherapy.

Another notable finding in our study was the rarity of mutations in *TET2* and other genes involved in DNA methylation pathway. Our cohort included 13 MDS/MPN cases and 4 AML cases with a prior history of MDS/MPN. *TET2* mutations are highly prevalent in cases of aCML, CMML and MDS/MPN unclassifiable (41%, 62% and 26% respectively) as shown by several authors, although these studies have not looked into cases with isolated i(17q) abnormality in particular [14–25].

A limitation of our study is the low detection sensitivity of Sanger sequencing used for assessment of *SRSF2* and *SETBP1*. The Sanger sequencing technique has a detection sensitivity of 10–20%, compared to NGS-based assay (5%). Hence, the mutational frequencies of *SRSF2* and *SETBP1* may be underestimated in our results. A strength of this study is that it is the first study to perform sequential mutational analysis of patients over the course of karyotypic progression, from diploid to i(17q). The findings in these 5 patients suggest that *ASXL1*, *SRSF2* and *RAS* pathway gene mutations occur prior to i(17q). In a subset of cases, *SETBP1* mutation may be associated with the development of i(17q) abnormality in these neoplasms. Interestingly, we found 1 patient with a history of primary myelofibrosis who transiently acquired i(17q) abnormality. This abnormality was present in 4/20 and 1/20 metaphases (confirmed by uniallelic *TP53* by FISH), at two different time points, 6 months apart. Bone marrow morphology showed myelofibrosis associated with trilineage dysplasia and monocytosis. Subsequently, the i(17q) abnormality disappeared (also confirmed by array based comparative hybridization and FISH studies), and the patient acquired del(3)(q12q24) and trisomy 8. The mutation profile remained the same. NGS sequencing based mutation analysis at 3 different time points showed mutations in *JAK2, ASXL1* and *KRAS; SRSF2* and *SETBP1* were wild-type (Figure 2). These results support that i(17q) abnormality is associated with mutated *SRSF2* and *SETBP1*.

Our results provide insights into the molecular consequences of i(17q), which leads to the obligatory loss of a single *TP53* allele located at 17p13.1 [7]. We have confirmed that *TP53* mutations, even at low allelic frequencies, are exceedingly rare in myeloid neoplasms with isolated i(17q) using next-generation sequencing based mutation analysis. This finding suggests involvement by mechanisms other than *TP53* mutation-induced genomic instability [1, 3, 9, 10]. Alternatively, *TP53* dysfunction may be the result of copy number changes or alterations in RNA and protein expression of other molecules of *TP53* pathway that have not been explored. At the same time, a potential role for several hematopoiesis-related candidate genes on 17q has not been explored. These genes include *NF1, RARA, G-CSF*, *MPO*, *ERBB2*, and *BRCA1* genes and miRNAs. Of note, *SRSF2* located at 17q25.1 was mutated in 55% cases in this study. *SRSF2* P95 mutation is a gain-of-function mutation that causes alterations in the binding affinity of *SRSF2* to a number of target pre-mRNA transcripts thereby affecting alternative splicing and gene expression [26]. Sequential mutational analysis suggests that *SRSF2* mutation, along with *ASXL1*, occurs early during the disease, and is present at the time the karyotype is diploid. In these cases, the formation of i(17q) leads to a higher dosage of mutant *SRSF2* and alterations of gene expression on a larger scale. The combination of *SRSF2* and *ASXL1* mutations, together with mutations in *SETBP1* may contribute to the dismal outcome.

In summary, we have performed a comprehensive gene mutational analysis in the largest series of myeloid neoplasms with isolated i(17q) abnormality to date. Our results show a high frequency of mutations in *SRSF2*, *SETBP1, ASXL1* and *NRAS* genes. In cases where isolated i(17q) abnormality was acquired during the disease course, *SRSF2* and *ASXL1* mutations preceded i(17q) detection, whereas *SETBP1* mutations were associated with i(17q). The acquisition of i(17q) abnormality and the presence of these adverse molecular markers may contribute to the poorer outcome of these patients. *SRSF2* and *RAS* pathway gene mutations may be potential therapeutic targets for aggressive management of these neoplasms.

## MATERIALS AND METHODS

### Patient cohort

This study was approved by the institutional review board. Informed consent was obtained from all patients. We retrieved all cases of myeloid neoplasm with i(17q) as the primary cytogenetic abnormality from our database from 1998–2014. Clinical data was collected from the medical records. All relevant pathology material including peripheral blood, bone marrow smears and biopsy sections, and immunocytochemical stains were reviewed. All cases were classified according to the 2008 WHO classification.

### Cytogenetic analysis

Conventional cytogenetic studies were performed on metaphase cells prepared from bone marrow aspirate smears using standard techniques. The results were reported using the International System for Human Cytogenetic Nomenclature (2009 and 2013).

### Genetic analysis

We performed amplicon-based next-generation sequencing (NGS) targeting the coding regions of a panel of 28 genes implicated in myeloid neoplasms using MiSeq platform (Illumina, San Diego, CA) on archived DNA extracted from fresh bone marrow aspirate samples. We used 250 ng of DNA to prepare the genomic library. The genes included in this panel are as follows: *ABL1, ASXL1, BRAF, DNMT3A, FGFR, EZH2, FLT3, GATA1, GATA2, HRAS, IDH1, IDH2, IKZF2, JAK2, KIT, KRAS, MDM2, MLL, MPL, MYD88, NOTCH1, NPM1, NRAS, PTPN11, RUNX1, TET2, TP53*, and *WT1*. Following successful library generation and purification, equal quantities of DNA from each sample were used for multiplex paired-end sequencing on MiSeq personal genome sequencer using the MiSeq Reagent Kit v2 (500 cycles). Human genome build 19 (hg19) was used as the reference for sequence alignment. MiSeq Reporter Software 2.2 and Integrative Genomics Viewer (IGV) were used for variant calling and visualization respectively. Successful sequencing was indicated by a Q30 score of more than 85%. For this study, variants with more than 5% allelic frequency with a minimum coverage of 250X in both directions were included. Since matched normal tissue was not tested, data from literature and COSMIC database was used to infer somatic nature. Single nucleotide polymorphisms listed in dbSNP 137 and 138 and 10K genome project were excluded. The functional significance of all variants was analyzed using PolyPhen-2 website (http://genetics.bwh.harvard.edu/pph2/); only variants that resulted in “probably damaging” were included. Selected variants were confirmed using an alternate platform, such as Sanger sequencing, pyrosequencing or capillary electrophoresis (data not shown). Mutation analyses of 5 additional genes that are not included in the NGS panel (*SETBP1*, *CEBPA, MDM4*, and splicing factor genes *SRSF2*, *SF3B1* and *U2AF1)* were performed by Sanger sequencing. In 25 cases, *FLT3* mutational analysis for internal tandem duplication was also performed by capillary electrophoresis.

### Statistical analysis

Frequencies and percentages were calculated for categorical variables, and means and standard deviation were calculated for continuous variables. Fisher's exact test was used to assess the association between categorical variables and mutation rate (not corrected for multiple testing). All statistical analyses were performed using SAS 9.3 for Windows.

## SUPPLEMENTARY MATERIALS FIGURES AND TABLES


