# Supplementary material for: Myeloid neoplasms with isolated isochromosome 17q demonstrate a high frequency of mutations in SETBP1, SRSF2, ASXL1 and NRAS
Source: Oncotarget. 2016 Feb 12;7(12):14251–8. doi: 10.18632/oncotarget.7350 (PMC4924712; doi:10.18632/oncotarget.7350)
Supplement: Supplementary file 1 [file oncotarget-07-14251-s001.pdf]

Myeloid neoplasms with isolated isochromosome 17q demonstrate a high frequency of mutations in *SETBP1*, *SRSF2*, *ASXL1* and *NRAS*

Supplementary Materials

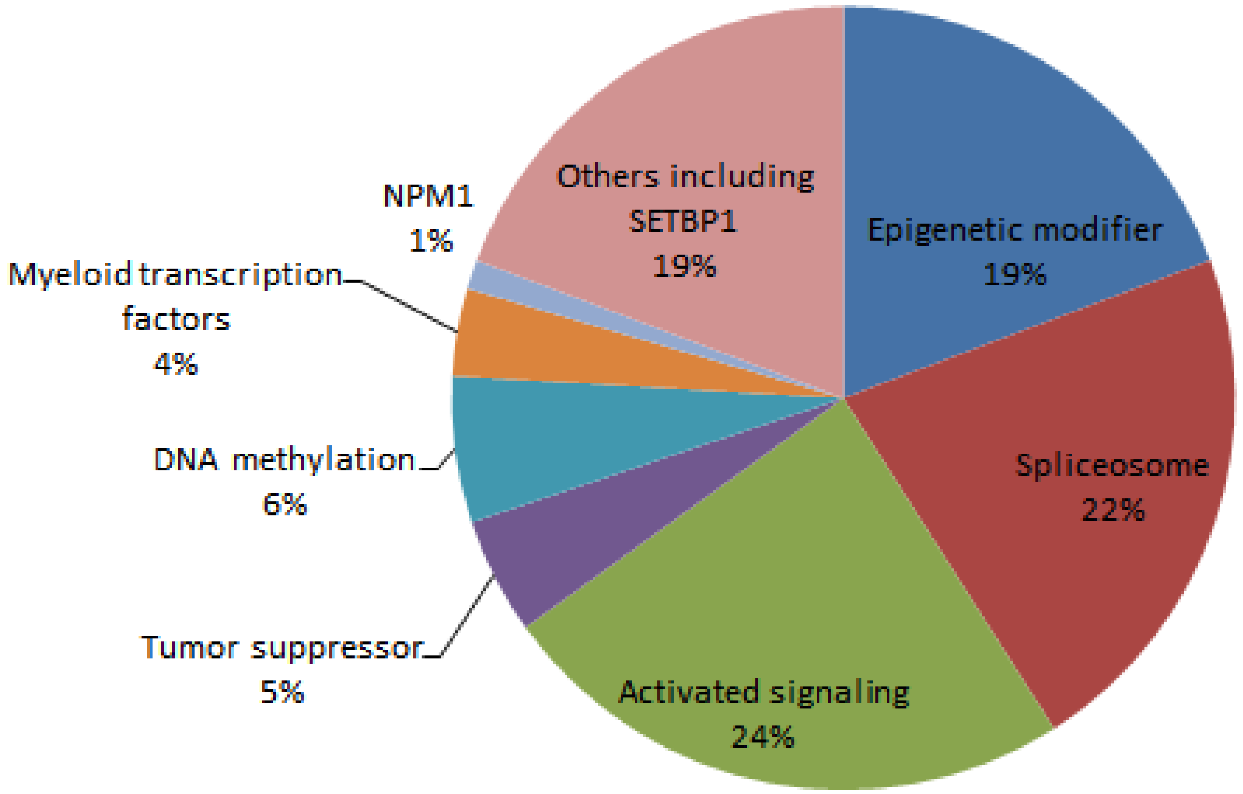

Supplementary Figure S1: Distribution of mutations into various biologic functional categories in myeloid neoplasms with i(17q) shows prominent involvement of genes of activating signaling pathway (24%, mostly in genes directly involved in *RAS* signaling), followed by spliceosome (22%, mostly *SRSF2*), epigenetic modifier genes (19%, *ASXL1*) and *SETBP1* (19%).

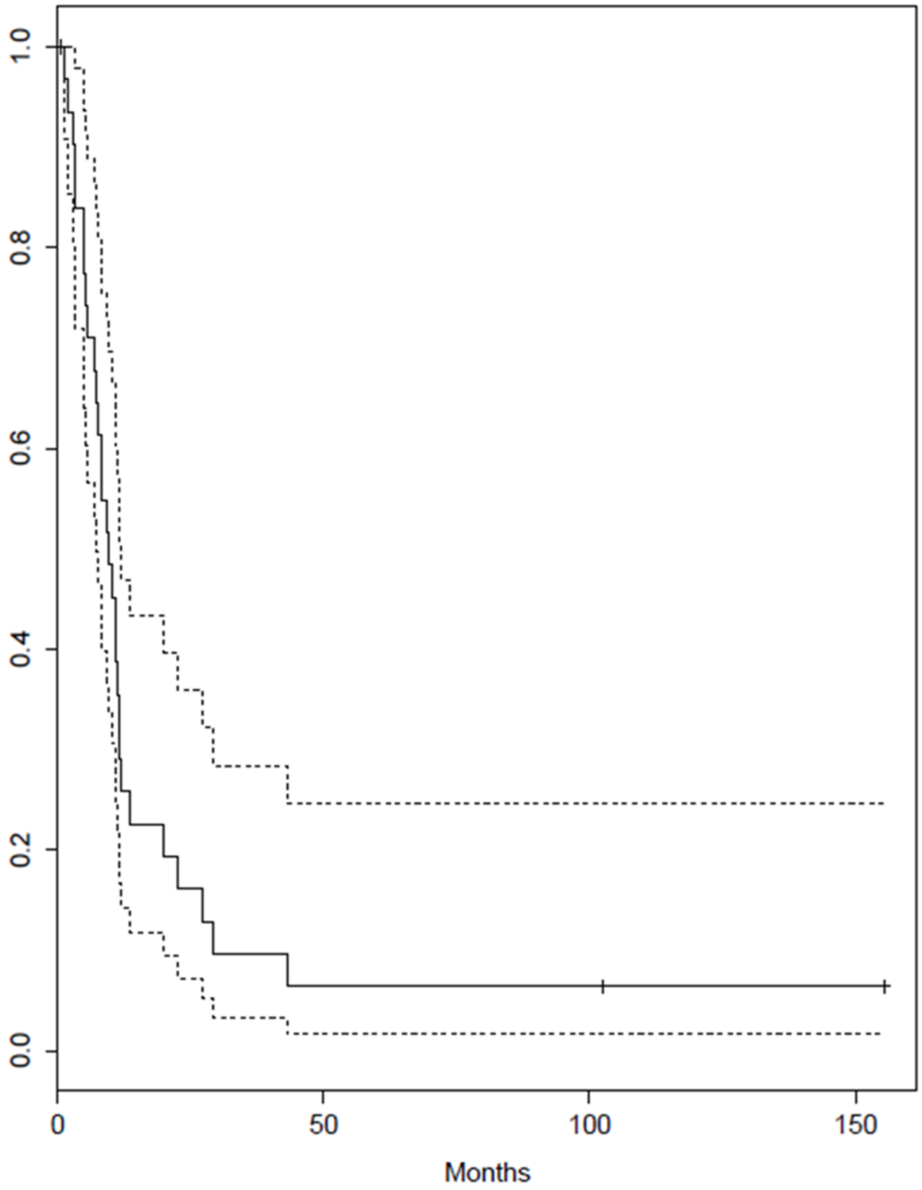

Supplementary Figure S2: Kaplan-Meier curve showing overall survival from the onset of i(17q) abnormality. The upper and lower dotted lines represent 95% CI.

Supplementary Table S1: Whole-exome sequencing results on 3 cases of myeloid neoplasms associated with isolated i(17q) abnormality

Case	Diagnosis	Mutation	VAF (%)
7	CMML, 8% blasts	*ASXL1* p. Q708X	43.609
		NRAS p.G12R	40.4762
		SETBP1 p.G870S	49
		SRSF2 p.P95H	57.7778
9	CMML, 18% blasts	*PTPN11* p. D61G	42.1053
		SETBP1 p.G870S	46.988
		SRSF2 p.P95H	60
		ETV6 p.Q255X	48.2759
31	AML arising from MDS, post allogeneic stem cell transplant	*ASXL1* p.Q592X	35.3261
		SETBP1 p. G870S	29.1005
		TP53 p. R249G	24.3243
		U2AF1 p. 157P	24.5509
		NF1 p.Q2637X	27.4112
		EGFR essential splice site	25.9494

Supplementary Table S2: A list of mutations identified by 28-gene next-generation sequencing (NGS) based analysis and Sanger sequencing in all patients with the associated variant allele frequencies (for genes studied by NGS)

Case #	Diagnosis	Mutation	Variant Allele Frequency	Number of Mutations
1	MDS	*ASXL1* p.G646fs*	39.2	2
		RUNX1 p.R166G	35.1	
2	aCML	*ASXL1* R693X	25.16	4
		KRAS G12R	NA	
		SETBP1 p.P187N	NA	
		SRSF2 p.P95R	NA	
3	aCML	*ASXL1* p.A1016fs	52.09	2
		SRSF2 p.P95R	NA	
4	CMML-1	*ASXL1* p.A634fs	16.8	5
		JAK2 p.V617F	49.28	
		NRAS p.G12D	42.9	
		SETBP1 p.D868N	NA	
		SRSF2 p.P95R	NA	
5	CMML-1	*ASXL1* p.A634fs	22.45	5
		EZH2 p.R690H	29.15	

		IDH2 p.R140Q	10.54	
		JAK2 p.V617F	50.71	
		SRSF2 p.P95L	NA	
6	CMML-1	*ASXL1* p.L775*	49.2	5
		GATA2 p.K390E	51.8	
		NRAS p.G12D	39.1	
		NRAS p.G12S	9.4	
		SETBP1 p.G870S	NA	
		SRSF2 p.P95R	NA	
7	CMML-1	*ASXL1* p. Q708X	43.6	4
		NRAS p.G12R	40.5	
		SETBP1 p.G870S	49	
		SRSF2 p.P95H	57.78	
8	CMML-2	*ASXL1* p.R1068*	47.7	4
		PTPN11 p.F71L	47.38	
		SETBP1 p.D868N	NA	
		SRSF2 p.P95R	NA	
9	CMML-2	*PTPN11* p. D61G	42.11	3
		SETBP1 p.G870S	46.99	
		SRSF2 p.P95H	60	
10	MDS/MPN U	*SETBP1* p.D868N	NA	2
		SRSF2 p.P95R	NA	
11	MDS/MPN U	*SRSF2* p.P95H	NA	1
12*	MDS/MPN U	*ASXL1* p.M837_ K838delinsI*	30.7	4
		EGFR p.G796S	11.4	
		ABL1 p.L248Q	12.40	
		SRSF2 p.P95R	NA	
13	MDS/MPN U	*ASXL1* insGfs	10.28	2
		HRAS p.E143K	53.86	
14	MDS/MPN U	*NRAS* p.Q61R	5.44	1
15	AML MRC	*ASXL1* p.A634fs	19.69	4
		NRAS p.G12D	16.32	
		SETBP1 p.G870S	NA	
		SRSF2 p.P95H	NA	
16	AML MRC	*FLT3* p.D835H	30.1	2
		WT1 p.V362fs	56.94	
17	AML MRC	*NRAS* p.G12D	45.84	3
		SETBP1 p.D868N	NA	
		U2AF1 p.Q157P	NA	
18	AML MRC	*NRAS* p.G12D	49.11	3
		NRAS p.G12D	49.2	
		SETBP1 p.G870S	NA	

		SRSF2 p.P95H	NA	
19	AML M1	*DNMT3A* p.Q415X	6.11	3
		IDH1 p.R132C	10.09	
		FLT3 ITD	NA	
20	AML MRC	*NRAS* p.G12D	43.76	2
		SETBP1 p.D868N	NA	
21	AML MRC	*KIT* p.E85*	23.8	1
22	AML M4	*ASXL1* p.L775*	44.5	4
		PTPN11 p.A72V	45.4	
		SETBP1 p.D874N	NA	
		SRSF2 p.P95H	NA	
23	AML M2	*ASXL1* p.L1170*	72.3	4
		ASXL1 p.G1198R	54.9	
		TP53 p.P190L	93.2	
		KRAS p.K117N	10.5	
		U2AF1 p.Q157P	NA	
24	Secondary AML arising from MDS/MPN (CMML)	*ASXL1* p.H630_I641del	34.82	3
		KRAS p.T58R	7.1	
		SETBP1 p.D868H	NA	
25	Secondary AML arising from MDS/MPN	*NRAS* p.G12S	38.9	5
		ASXL1 p.C975*	9.1	
		TET2 p.L1212*	49.6	
		TET2 p.H682fs*	47.6	
		SETBP1 p.G870S	NA	
		SRSF2 p.P95L	NA	
26	Secondary AML arising from MDS	*SETBP1* p.D868N	NA	1
27	Secondary AML arising from MDS/MPN	*FLT3* ITD	NA	3
		SETBP1 p.D868N	NA	
		SRSF2 p.P95R	NA	
28	Secondary AML arising from MDS/MPN (CMML)	*NRAS* p.G12D	44.1	1
29	Therapy related AML	*RUNX1* p.S114_S115insT	15.76	1
30	Therapy related AML	*NPM1* p.W288fs*	21.54	4
		PTPN11 p.G503R	22.15	
		IDH2 p.R140Q	22.75	
		DNMT3A p.R882H	14.56	
31	AML, cannot be classified	*ASXL1* p.Q592*	23.38	4
		TP53 p.R249G	32.4	
		SETBP1 p.G870S	29.10	

		U2AF1 p.Q157P	24.60	
32	Post-essential thrombocythemia myelofibrosis	*TET2* p.L703*	11.49	3
		ASXL1 p.G646fs*11	5.59	
		TP53 p.R273H	5.73	
